# Mesolimbic leptin signaling negatively regulates cocaine-conditioned reward

**DOI:** 10.1038/tp.2016.223

**Published:** 2016-12-06

**Authors:** M Shen, C Jiang, P Liu, F Wang, L Ma

**Affiliations:** 1 State Key Laboratory of Medical Neurobiology, School of Basic Medical Sciences and Institutes of Brain Science, and the Collaborative Innovation Center for Brain Science, Fudan University, Shanghai, China

## Abstract

The regulatory mechanisms underlying the response to addictive drugs are complex, and increasing evidence indicates that there is a role for appetite-regulating pathways in substance abuse. Leptin, an important adipose hormone that regulates energy balance and appetite, exerts its physiological functions via leptin receptors. However, the role of leptin signaling in regulating the response to cocaine remains unclear. Here we examined the potential role of leptin signaling in cocaine reward using a conditioned place preference (CPP) procedure. Our results showed that inhibition of leptin signaling by intracerebroventricular infusion of the leptin receptor (LepR) antagonist SMLA during cocaine conditioning increased the cocaine-CPP and upregulated the level of dopamine and its metabolites in the nucleus accumbens (NAc). We then selectively knocked down the LepR in the mesolimbic ventral tegmental area (VTA), NAc core and central amygdala (CeA) by injecting *AAV-Cre* into *Lepr*^*flox/flox*^ mice. LepR deletion in the VTA increased the dopamine levels in the NAc and enhanced the cocaine-conditioned reward. LepR deletion in the NAc core enhanced the cocaine-conditioned reward and impaired the effect of the D2-dopamine receptor on cocaine-CPP, whereas LepR deletion in the CeA had no effect on cocaine-CPP but increased the anxiety level of mice. In addition, prior exposure to saccharin increased *LepR* mRNA and STAT3 phosphorylation in the NAc and VTA and impaired cocaine-CPP. These results indicate that leptin signaling is critically involved in cocaine-conditioned reward and the regulation of drug reward by a natural reward and that these effects are dependent on mesolimbic LepR.

## Introduction

The hormones of the neuroendocrine system modulate and command perception, cognition and the emotional process.^1^ They regulate multiple physiological and pathological states, such as eating and digestion, exploration and survival, and addiction and anxiety.^2^ Hormones influence motivation and reward through changing the release of different neurotransmitters in substance abuse and eating disorders.^3^ Increasing evidence shows that appetite-regulating hormones such as glucagon-like peptide, ghrelin and leptin are involved in the development processes of substance abuse.^4, 5^ Consumption of palatable food and addictive drugs can promote continuous overfeeding or drug-seeking behaviors through eliciting similar hormone-mediated neuroadaptive changes within brain reward circuitry and stress systems,^6^ indicating that there might be shared neuronal circuits or molecular mechanisms in food and addictive rewards.

Leptin, an adipose-derived hormone,^7^ affects food intake, body weight,^8^ energy balance,^9^ emotional behaviours^10^ and natural rewarding effects.^11^ Leptin inhibits the rewarding effects of food via mesolimbic reward circuits as well as the hypothalamus.^12^ Previous work has examined the influence of abnormal systemic leptin levels on addictive behaviors by genetic manipulation or the use of pathological animal models.^13, 14^ The systemic injection of leptin^15^ and the direct infusion of leptin or the leptin receptor (LepR) agonist into the ventral tegmental area (VTA) attenuates the rewarding effects of cocaine.^16^ However, the functional role of leptin signaling within mesolimbic brain regions in regulating the response to addictive drugs still needs to be thoroughly elucidated.

Leptin exerts its biological effects via binding to the functional LepRs, which are widely expressed in the brain.^17^ Previous results have shown that specific deletion of LepR in glutamatergic neurons located in the hippocampus and prefrontal cortex causes a depressive-like phenotype,^18, 19^ while ablation of the LepR in dopamine neurons results in a robust anxiogenic phenotype,^20^ indicating that ablation of the LepR elicits long-term changes in different brain circuits and modulates different behavior phenotypes. However, there may be deficiencies or adaptions during the development of neural circuits in those conditioned knockout mice. Studies examining the effects of acute manipulation of endogenous leptin signaling on drug-reward are lacking.

In this study, we inhibited LepR-mediated signaling in several regions in the mesolimbic system by infusion of the LepR antagonist superactive mouse leptin antagonist (SMLA) or injection of AAV-Cre into the VTA, nucleus accumbens (NAc) or central amygdala (CeA) to selectively deplete LepRs in *Lepr*^*flox/flox*^ mice. We then investigated the role of leptin signaling in cocaine-conditioned place preference (CPP) and cocaine-induced dopamine release. In addition, the role of mesolimbic LepR signaling on the effect of a natural reward on cocaine-CPP was also assessed.

## Materials and methods

### Animals and housing

B6 *ob/ob* mice (strain name: B6.Cg-Lep^ob^/J) were obtained from the Model Animal Research Center of Nanjing University. *Lepr*^*flox/flox*^ mice, in which exon 1 of the *Lepr* gene is floxed, were obtained from The Jackson Laboratory (Bar Harbor, ME, USA) (strain name: B6.129P2-Lepr^*tm1Rck*^*/J*). All mice were bred onto the C57BL/6J background. *Lepr*^*flox/flox*^ mice and their wild-type (WT) littermates were obtained from self-crossing of *Lepr*^flox/+^ (heterozygous) mice. Mice were housed in groups on a 12 h light/dark cycle with food and water available *ad libitum* except for in the special case noted below. Genotypes were determined by PCR of mouse tail DNA samples. All animal treatments were strictly in accordance with the National Institutes of Health Guide for the Care and Use of Laboratory Animals and were approved by the Animal Care and Use Committee of the School of Basic Medical Sciences, Fudan University. Male mice, 8–10 weeks old, were used for behavioral tests.

### Cannula implantation and microinjection

Mice anesthetized with choral hydrate were placed in a mouse stereotactic instrument (Stoelting, Kiel, WI, USA) and implanted with cannula guides (Plastics one, Roanoke, VA, USA) over the lateral ventricles, VTA, or NAc core. The cannulas were secured to the mice with dental cement. The intended stereotaxic coordinates were as follows: lateral ventricles: anterior–posterior (AP) −0.2 mm; medial–lateral (ML) ±1.0 mm; dorsal–ventral (DV) −2.2 mm; VTA: AP −3.2 mm; ML ±0.5 mm; DV −4.4 mm; and NAc core: AP +1.4 mm; ML ±1.2 mm; DV −4.3 mm. All mice were given at least 7 days to recover before behavioral experiments.

SMLA (BioSource, San Diego, CA, USA) mimics the Asp-23 mutation of leptin that exhibits a high affinity for the leptin receptor and antagonistic activity *in vitro* and *in vivo*. SMLA was dissolved in artificial cerebrospinal fluid (ACSF) (126 mM NaCl, 26 mM NaHCO_3_, 1.2 mM NaH_2_PO_4_, 3 mM KCl, 2.4 mM CaCl_2_, 1.3 mM MgCl_2_, 10 mM d-glucose) to 250 ng μl^−1^, and intracerebroventricular (i.c.v.) administration of SMLA through the cannula was performed at a total volume of 2 μl at a slow rate (0.2 μl min^−1^). Mice received microinjection of SMLA (500 ng) or ACSF as the vehicle (2 μl) 30 min before or after cocaine conditioning. D2-dopamine receptor (D2R) agonist bromocriptine (Tocris Bioscience, Bristol, UK) was dissolved in ACSF to 250 ng μl^−1^, and mice received a bilateral microinjection of 2 μl bromocriptine solution into the NAc core at a slow rate (0.2 μl min^−1^) 30 min before cocaine conditioning.

### Viral infection

Male adult *Lepr*^*flox/flox*^ mice and their WT littermates anesthetized with choral hydrate were placed in a mouse stereotactic instrument. Microinjections were performed using custom-made injection needles (33-gauge) connected to a 10 μl Hamilton syringe. Each brain nucleus was injected with 0.5 μl of purified and concentrated AAV2/5 (5 × 10^12^ IU ml^−1^) encoding *CAG-eGFP-T2A-Cre* at a slow injection rate (0.1 μl min^−1^). The intended stereotaxic coordinates were as follows: NAc core: AP +1.4 mm; ML ±1.2 mm; DV −4.3 mm; VTA: AP −3.2 mm; ML ±0.5 mm; DV −4.4 mm; and CeA: AP −1.3 mm; ML ±2.7 mm; DV −4.6 mm. All mice were given at least 14 days to recover before behavioral experiments, and the virus infections and the knockdown efficiency were checked 14 days after the injection by immunostaining. The histology slides were examined blindly to quantify the expression of LepR in EGFP positive cells.

### High-performance liquid chromatography

The levels of monoamine neurotransmitters, including norepinephrine (NA), dopamine (DA) and 5-hydroxytryptamine (5-HT), and their respective metabolites, 3,4-hydroxyphenylacetic acid (DOPAC), homovanillic acid (HVA), and 5-hydroxyindoleacetic acid (5-HIAA), were detected 30 min after cocaine (15 mg kg^−1^ i.p.) injection using high-performance liquid chromatography (HPLC) as previously described.^21^ Within 5 min of the cocaine injection, the NAc and amygdala of mice were dissected in iced phosphate-buffered saline by a vibratome (Microm HM 650V, Thermo Fisher Scientific, Waltham, MA, USA), according to the stereotaxic coordinates listed below: NAc: from Bregma 1.54 to 0.54 mm and amygdala (AMG): from Bregma −1.06  to −2.06 mm. The NAc and AMG were then homogenized in 100 μl ice-cold 0.1 M perchloric acid containing 10 μM ascorbic acid, 0.1 mM EDTA disodium salt and 0.02 μM 3,4-dihydroxybenzylamine. The homogenates were centrifuged at 20 000 *g* for 10 min at 4 °C, and the supernatants were collected for analysis. An HPLC system with electrochemical detection (UltiMate 3000 system; Thermo Fisher Scientific) was used, and the supernatants were injected onto an Acclaim C18 column (2.2 μm, 2.1 × 100 mm; Thermo Fisher Scientific) at 38 °C. Separations were performed at a flow rate of 0.2 ml min^−1^ using a mobile phase of phosphate buffer, containing 0.05 mM EDTA, 1.7 mM orthosilicic acid, 90 mM Na_2_HPO_4_, 50 mM citric acid and 5% acetonitrile. The data were collected and analyzed by Chromeleon chromatography workstation (Thermo Fisher Scientific).

### Cocaine- or palatable-food-induced CPP

CPP induced by cocaine hydrochloride (Qinghai Pharmaceutical Firm, Qinghai, China) was blindly performed by an investigator using a two-chamber apparatus (Med-Associates, St Albans, VT, USA) with distinct tactile environments to maximize contextual differences. One chamber of the box had a wire mesh floor, while the other chamber had a grid rod floor. A manual guillotine door (7 × 5 cm) separated the two chambers. On Day 1, mice were placed in one of the chambers and allowed to freely explore the entire apparatus for 20 min (pre-test). The mice staying in one chamber for more than 13 min were excluded from the experiment. Mice were randomized into two groups by tossing a coin to receive vehicle or drugs. On days 2, 3 and 4, mice were given an intraperitoneal injection of cocaine (15 mg kg^−1^, i.p.) in the morning and confined to one of the chambers (drug-paired) for 30 min, and in the afternoon, they received an i.p. injection of saline (an equivalent volume to that of cocaine) and were confined to the other chamber for 30 min (conditioning). The distance the mice traveled within the 30 min was recorded. On Day 5, mice were allowed to freely explore the entire apparatus for 20 min (test). The time spent in each chamber was recorded during the pre-test and test sessions. The CPP score was defined as the time (in seconds) spent in the cocaine-paired chamber minus the time spent in the saline-paired chamber. All mice used in the cocaine-CPP were satiated.

CPP induced by palatable food was assessed using an approach similar to that previously reported.^22, 23^ All mice were food-restricted for 6 h before being conditioned to chocolate-flavored pellets (20 mg, 5.5% fat, 18.4% protein, 59.1% carbohydrate, 3.6 kcal g^−1^, #F05301 Bio-serv, Flemington, NJ, USA) or chow food (6% fat, 18% protein, 58% carbohydrate, Xietong Organism, Jiangsu, China). Plates containing chocolate-flavored pellets and chow food were placed in the home-cages at least 1 week before behavior tests for adaptation. A two-chamber apparatus (each chamber was 13 × 26 cm) with distinct tactile environments to maximize contextual differences was used. A manual guillotine door separated the two chambers. One chamber had a white rhombus-patterned wall and black plastic floor, and the other consisted of a black striped wall and white floor with three 3 cm abrasive sticks. On Day 1, mice were placed in one of the chambers and allowed to freely explore the entire apparatus for 20 min (pre-test). On Days 2, 4, 6, 8 and 10, mice were confined to one chamber in the corner of which was placed a plate of chocolate-flavored pellets available *ad libitum* for 30 min, and on Days 3, 5, 7, 9 and 11, they were confined to the other chamber, which contained a plate of chow food for 30 min. On Day 12, mice were allowed to freely explore the entire apparatus for 20 min (test). The food intake was calculated as the weight of the palatable food or chow food before each session minus the weight recorded after the session. The time spent in each chamber was recorded during the pre-test and test sessions. The CPP score was defined as the time (in seconds) spent in the palatable food-paired chamber minus the time spent in the chow food-paired chamber.

### Saccharin operant task

Each operant behavior apparatus (21.6 cm length × 17.8 cm width × 12.7 cm height, Med-Associates) was equipped with a dim light source and a nose-poke hole (3.8 cm in diameter, 0.64 cm deep, 1.0 cm from the grid floor, Med-Associates) equipped with infrared photo-beams connected to a computer. In the center of the wall was a trough situated 2 cm from the grid floor from which liquid (0.1% saccharin (w/v) or water) was delivered when the photo-beam of the nose-poke hole was interrupted for at least 500 ms. A fixed-ratio 1 reinforcement schedule was initially applied for 12 consecutive days; that is, one nose-poke resulted in one delivery of liquid. Mice were divided into three groups by generating a random digit: water, acute saccharin and chronic saccharin, and each group of mice were confined to the apparatus for a 1-h test daily. The water group received a delivery of water after a nose-poke during the 1-h session for 12 days; the mice in the acute saccharin group received a delivery of water after a nose-poke for 11 days and, on Day 12, received a delivery of 0.1% saccharin as a reward after a nose-poke; the chronic saccharin group received a delivery of 0.1% saccharin after a nose-poke for 12 days. Cocaine CPP was blindly performed by an investigator.

### Statistical analysis

Seven to twelve mice per group were used for behavior tests, and 4–6 mice per group were used for HPLC and biochemistry studies. All data are presented as the mean±s.e.m. Student's *t*-test, two-way analysis of variance (ANOVA), or two-way repeated-measures analysis of variance (RM ANOVA) was used for statistical analysis. Tukey's *post hoc* analysis was subsequently performed after a two-way ANOVA or two-way RM ANOVA. *P<0.05, **P<0.01 and ***P<0.001.

## Results

### Leptin signaling in the brain negatively regulates cocaine-conditioned reward and cocaine-induced dopamine release in the NAc

**Figure 1:**
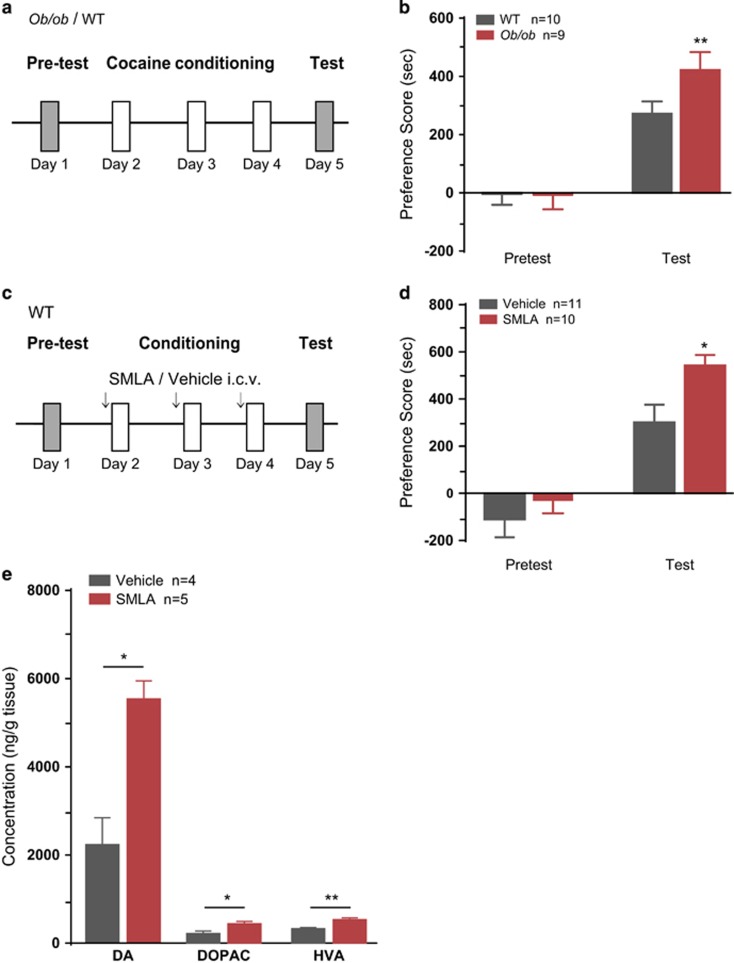
Downregulation of leptin signaling increases cocaine-CPP and dopamine levels in the NAc. (**a**) Schematic of the cocaine-CPP experimental schedule. WT and *Ob/ob* mice were tested for place preference on Day 1 and received cocaine (15 mg kg^−1^, i.p.) conditioning on Days 2, 3 and 4. On Day 5, mice were allowed to freely explore the entire apparatus for 20 min, and the time spent in each chamber was recorded. (**b**) Quantification of the place preference scores of WT and *Ob/ob* mice in the pre-test and test sessions. (**c**) Schematic of the experimental schedule. WT mice received an infusion of ACSF or SMLA (500 ng, i.c.v.) 30 min before conditioning sessions. (**d**) Quantification of the place preference scores in the pre-test and test sessions. (**e**) Concentrations of cocaine-induced monoamine neurotransmitters including DA, DOPAC and HVA in the NAc of mice receiving SMLA or Vehicle i.c.v. infusion 30 min before the cocaine injection were detected by HPLC. The data are presented as the mean±s.e.m. *P<0.05, **P<0.01. ACSF, artificial cerebrospinal fluid; CPP, conditioned preference place; HPLC, high-performance liquid chromatography; i.c.v., intracerebroventricular; NAc, nucleus accumbens; SMLA, superactive mouse leptin antagonist; WT, wild-type.

To explore the potential role of the endogenous leptin system in regulating drug reward, we examined cocaine (15 mg kg^−1^ i.p.)-induced CPP in *ob/ob* mice (systemic knockout of leptin) and their WT littermates (Figure 1a). The WT and *ob/ob* mice were given an injection of cocaine and trained to associate the drug with one chamber daily for 3 days. In the test session, both groups showed significant preference for the cocaine-paired chamber. *ob/ob* mice spent significantly more time in the cocaine-paired chamber compared with their WT littermates (two-way ANOVA, treatment × genotype: F(1, 17)=4.240, *P*=0.055. WT vs *Ob/ob* within test, *P*=0.009; Figure 1b). Leptin exerts its biological effects via activating the functional LepRs, which are widely expressed in the central nervous system.^24^ To assess the role of LepR signaling in the central nervous system in cocaine-conditioned reward, we tested the effects of i.c.v. infusion of a LepR antagonist, SMLA, on the acquisition of cocaine-CPP. Adult C57 WT mice received microinjection of vehicle or SMLA 30 min before or after the cocaine-context-conditioning period (Figure 1c and Supplementary Figure S1A). The SMLA-treated mice spent more time in the cocaine-paired chamber than the vehicle-treated individuals (Figure 1d: two-way ANOVA, treatment × genotype: F(1, 19)=1.572, *P*=0.218. Vehicle vs SMLA within test, *P*=0.019; S1B: treatment × genotype: F(1, 14)=1.684, *P*=0.215. Vehicle vs SMLA within test, *P*=0.022; Figure 1d and Supplementary Figure S1B). The cocaine-induced monoamine neurotransmitter levels in the NAc after SMLA i.c.v. administration were examined. The SMLA-treated mice exhibited significantly increased levels of dopamine and its metabolites in the NAc (Student's *t*-test, DA: *t*(7)=4, *P*=0.0023; DOPAC: *t*(7)=2.875, *P*=0.0238; HVA: *t*(7)=3.787, *P*=0.0068; Figure 1e), while the concentrations of norepinephrine (NE), 5-hydroxytryptamine (5-HT) and its metabolites 5-hydroxyindoleacetic acid (5-HIAA) (NE: *t*(7)=0.4596, *P*=0.6597; 5-HT: *t*(7)=1.090, *P*=0.3120; 5-HIAA: *t*(7)=1.049, *P*=0.3289) in the NAc were not changed (Supplementary Figure S2A). These results indicate that leptin signaling negatively regulates cocaine-conditioned reward and affects mesolimbic dopamine system.

### Leptin signaling in the VTA negatively regulates cocaine-conditioned reward

**Figure 2:**
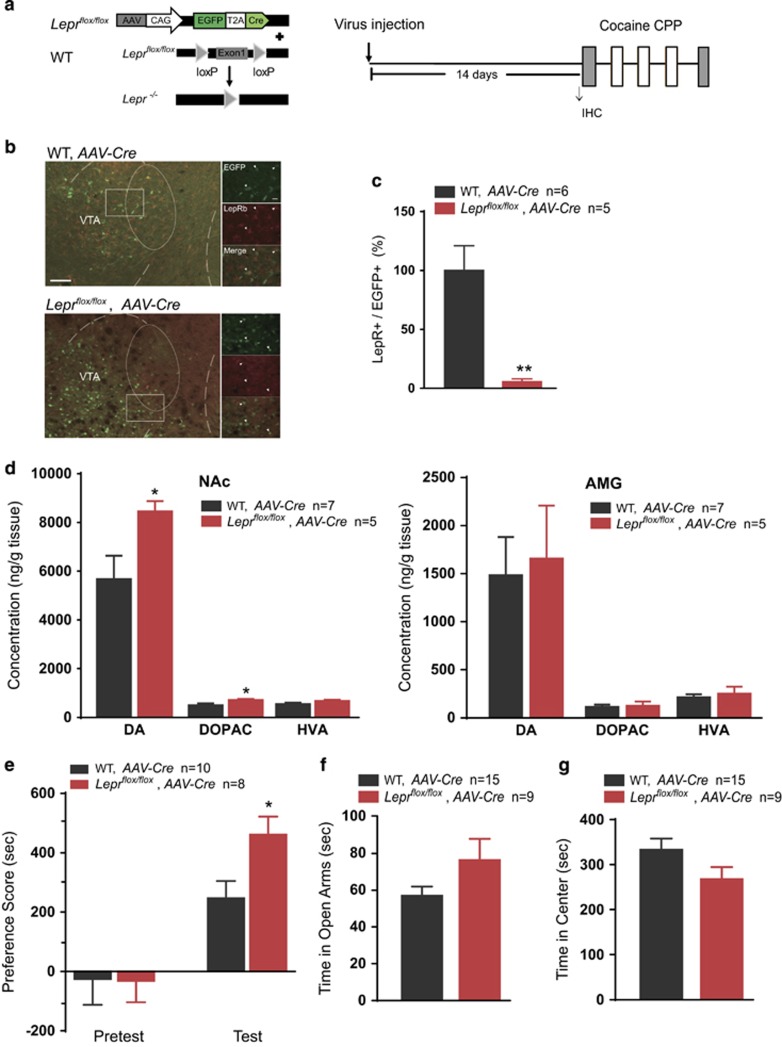
Leptin signaling in the VTA negatively regulates cocaine-conditioned reward and dopamine levels in the NAc. (**a**) Schematic drawing of the AAV*-CAG-EGFP-T2A-Cre* (left) and the experimental schedule of immunohistochemistry (IHC) and behavior tests after virus injection (right). (**b**) Representative images of the AAV*-CAG-EGFP-T2A-Cre*-infected VTA from *Lepr*^*flox/flox*^ mice and WT littermates. EGFP: green; LepR: red; arrows indicate the EGFP^+^ cells. Scale bar, 100 μm (low-magnification images) and 20 μm (high-magnification images). (**c**) Quantification of the percentage of LepR-positive cells in the EGFP^+^ populations in the VTA of virus-infected *Lepr*^*flox/flox*^ mice and WT littermates. (**d**) Concentration of cocaine-induced monoamine neurotransmitters in the NAc (left) and AMG (right) of *Lepr*^*flox/flox*^ and WT mice. (**e**) Quantification of the place preference scores of AAV-Cre-injected *Lepr*^*flox/flox*^ and WT mice in the pre-test and test sessions. (**f**) Time spent in the open arms of the elevated plus maze (test in the *Lepr*^*flox/flox*^ and WT mice. (**g**) Time in the center zone of the open-field test in the *Lepr*^*flox/flox*^ and WT mice. The data are presented as the mean±s.e.m. *P<0.05, **P<0.01. AMG, amygdala; NAc, nucleus accumbens; VTA, ventral tegmental area; WT, wild-type.

Addictive drugs are thought to activate the reward circuitry in the VTA that projects to the NAc, amygdala, hippocampus and prefrontal cortex.^25^ To assess the effects of endogenous LepRs in the mesolimbic system on cocaine reward-associated behaviors, we bilaterally injected *AAV-CAG-eGFP-T2A-Cre* into the VTA of *Lepr*^*flox/flox*^ mice and their WT littermates 14 days before behavior tests (Figure 2a), and LepR protein expression levels were assessed by immunostaining (Figure 2b). *AAV-CAG-eGFP-T2A-Cre* injection significantly reduced the number of GFP and LepR-double positive cells in the VTA of *Lepr*^*flox/flox*^ mice compared with that in the WT littermates (Student's *t*-test, *t*(9)=4.033, *P*=0.0030; Figure 2c and Supplementary Figure S3A), indicating a marked knockdown of endogenous LepRs in the VTA. The cocaine-induced monoamine neurotransmitter levels in the downstream targets of the VTA, including the NAc and AMG, were examined. We found that the AAV-Cre-injected *Lepr*^*flox/flox*^ mice exhibited increased levels of dopamine in the NAc (Student's *t*-test, DA: *t*(10)=2.307, *P*=0.0438; DOPAC: *t*(10)=2.433, *P*=0.0347; HVA: *t*(10)=1.582, *P*=0.14448; NE: *t*(10)=0.4968, *P*=0.6301; 5-HT: *t*(10)=0.1010, *P*=0.9216; 5-HIAA: *t*(10)=0.6076, *P*=0.5570; Figure 2d left panel and Supplementary Figure S2B), while there was no significant difference in the AMG compared with that in WT littermates (Student's *t*-test, DA: *t*(10)=0.5371, *P*=0.6029; DOPAC: *t*(10)=0.4601, *P*=0.6553; HVA: *t*(10)=0.8738, *P*=0.4027; NE: *t*(10)=0.6575, *P*=0.5257; 5-HT: *t*(10)=0.1710, *P*=0.8676; 5-HIAA: *t*(10)=2.156, *P*=0.0565; Figure 2d right panel and S2C), suggesting the knockdown of LepRs in the VTA might lead to impaired function of dopaminergic axons innervating NAc.

*Lepr*^*flox/flox*^ mice spent significantly more time in the palatable food-paired chamber than the WT individuals (two-way ANOVA, treatment × genotype: F(1, 16)=1.959, *P*=0.181. WT vs *Lepr*^*flox/flox*^ within test, *P*=0.047) in the test session (Supplementary Figures S1C and D) after five sessions of conditioning, which was consistent with the previous studies that leptin inhibited food reward through its effects on the mesolimbic reward circuitry.^26, 27^

We then evaluated the effects of LepR knockdown in the VTA on cocaine-CPP. After 3 days of cocaine conditioning, both groups showed a preference for the cocaine-paired chamber, while *Lepr*^*flox/flox*^ mice spent more time in the cocaine-paired chamber than the WT individuals (two-way ANOVA, treatment × genotype: F(1, 17)=2.739, *P*=0.119. WT vs *Lepr*^*flox/flox*^ within test, *P*=0.032; Figure 2e). There was no significant difference between AAV-Cre-injected *Lepr*^*flox/flox*^ and WT mice in the time spent in the open arms of the elevated plus maze test (Student's *t*-test, *t*(25)=1.713, *P*=0.0991; Figure 2f). In the open-field test, there was no significant difference between *Lepr*^*flox/flox*^ mice and their WT littermates in the time spent in the center zone, entries into the center, or the total distance travelled (Student's *t*-test, time: *t*(22)=1.696, *P*=0.1040; entries: *t*(22)=0.1297, *P*=0.8980; total distance travelled: *t*(25)=0.4079, *P*=0.6869; Figure 2g, Supplementary Figures S4A and B). These data suggest that leptin signaling in the VTA might decrease the dopamine level in the NAc and negatively regulate cocaine-conditioned reward without affecting the anxiety level.

### Leptin signaling in the NAc core negatively regulates cocaine-conditioned reward and is critical for the function of the D2R

**Figure 3:**
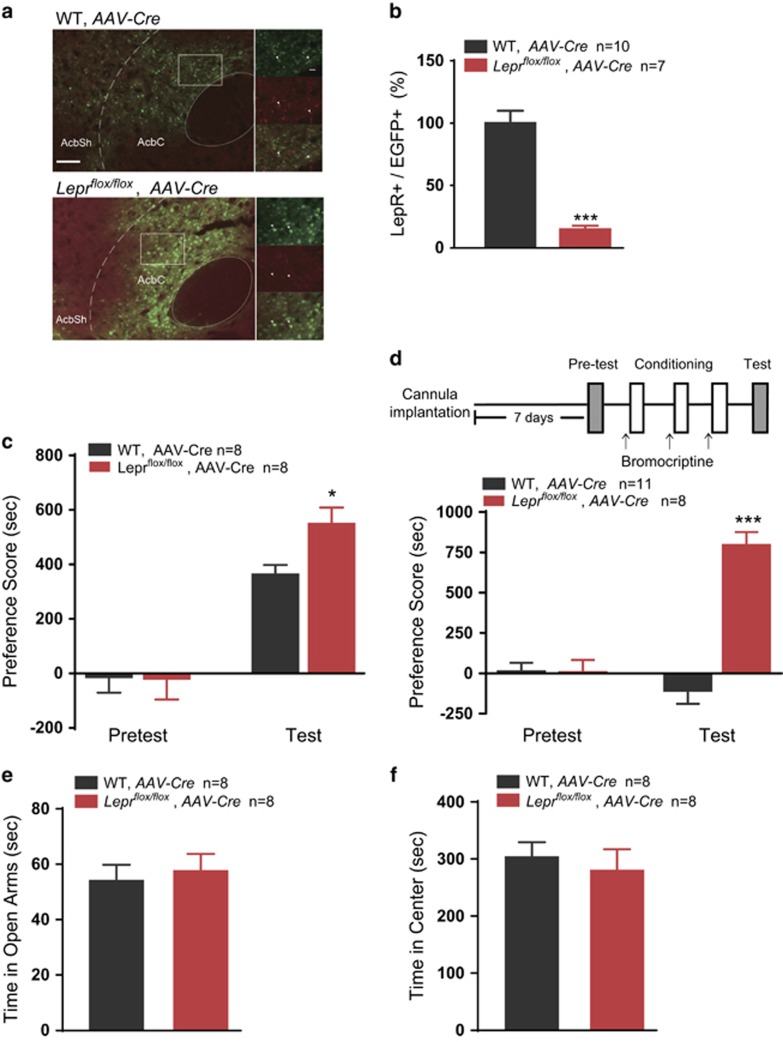
Leptin signaling in NAc Core negatively regulates cocaine-conditioned reward and is essential for D2R function. (**a**) Representative images of the AAV*-CAG-EGFP-T2A-Cre*-infected NAc from *Lepr*^*flox/flox*^ mice and WT littermates. (**b**) Quantification of the number of LepR-positive cells in the EGFP^+^ cells in the NAc core of virus-infected *Lepr*^*flox/flox*^ mice and WT littermates. EGFP: green; LepR: red; arrows indicate the EGFP^+^ cells. Scale bar, 100 μm (low-magnification images) and 20 μm (high-magnification images). (**c**) Quantification of the place preference scores of virus-infected *Lepr*^*flox/flox*^ and WT mice in the pre-test and test sessions. (**d**) Schematic of the experimental schedule for cocaine-CPP after cannula implantation (upper panel). Mice received bilateral infusion of the D2R agonist bromocriptine (500 ng) into the NAc core 30 min before cocaine conditioning, and the quantification of the place preference scores in the pre-test and test sessions is shown below. (**e**) Time spent in the open arms of the elevated plus maze test in *Lepr*^*flox/flox*^ and WT mice. (**f**) Time in the center zone of the open-field test in the *Lepr*^*flox/flox*^ and WT mice. The data are presented as the mean±s.e.m. *P<0.05, **P<0.01, ***P<0.001. CPP, conditioned preference place; D2R, D2-dopamine receptor; LepR, leptin receptor; NAc, nucleus accumbens; WT, wild-type.

To assess the effect of leptin signaling in the downstream brain regions of the VTA on cocaine-CPP, we bilaterally injected *AAV-CAG-eGFP-T2A-Cre* into the NAc core of *Lepr*^*flox/flox*^ mice and their WT littermates (Figure 3a), and the loss of the LepR in the NAc was evaluated by immunostaining (Student's *t*-test, *t*(15)=6.943, *P*<0.001; Figure 3b and Supplementary Figure S3B). *Lepr*^*flox/flox*^ mice injected with virus spent more time in the cocaine-paired chamber than their WT littermates (two-way ANOVA, treatment × genotype: F(1, 14)=2.671, *P*=0.124. WT vs *Lepr*^*flox/flox*^ within test, *P*=0.040; Figure 3c). It has been reported that leptin signaling increases D2R activity in the striatum.^28^ To assess whether NAc leptin signaling is involved in the D2R-dependent pathway, we injected the D2R agonist bromocriptine into the NAc core of AAV-Cre-infected mice 30 min before each conditioning session. The results showed that D2R activation eliminated cocaine-CPP in WT mice but not in *Lepr*^*flox/flox*^ mice (two-way ANOVA, treatment × genotype: *F*(1, 17)=42.617, *P*<0.001. WT vs *Lepr*^*flox/flox*^ within test, *P*<0.001) in the test session (Figure 3d), suggesting that LepRs might be essential for the inhibitory effects of cocaine-CPP induced by D2R-positive neurons of the NAc.

The time spent in the open arms in the elevated plus maze experiments (Student's *t*-test, time in the open arms: *t*(16)=0.4077, *P*=0.6889; Figure 3e) and the time in the center zone, the entries into center, and the total distance travelled in the open-field test also showed no significant differences between AAV-Cre-injected *Lepr*^*flox/flox*^ mice and their WT littermates (time in the center zone: *t*(18)=0.5111, *P*=0.6155; entries: *t*(18)=0.4255, *P*=0.6755; total distance travelled: *t*(18)=0.1627, *P*=0.8726; Figure 3f, Supplementary Figures S4C and D), suggesting that leptin signaling in the NAc negatively regulates cocaine-conditioned reward and D2R function while having no effects on anxiety or locomotor activity.

### Leptin signaling in the CeA is involved in anxiety but not cocaine-conditioned reward

**Figure 4:**
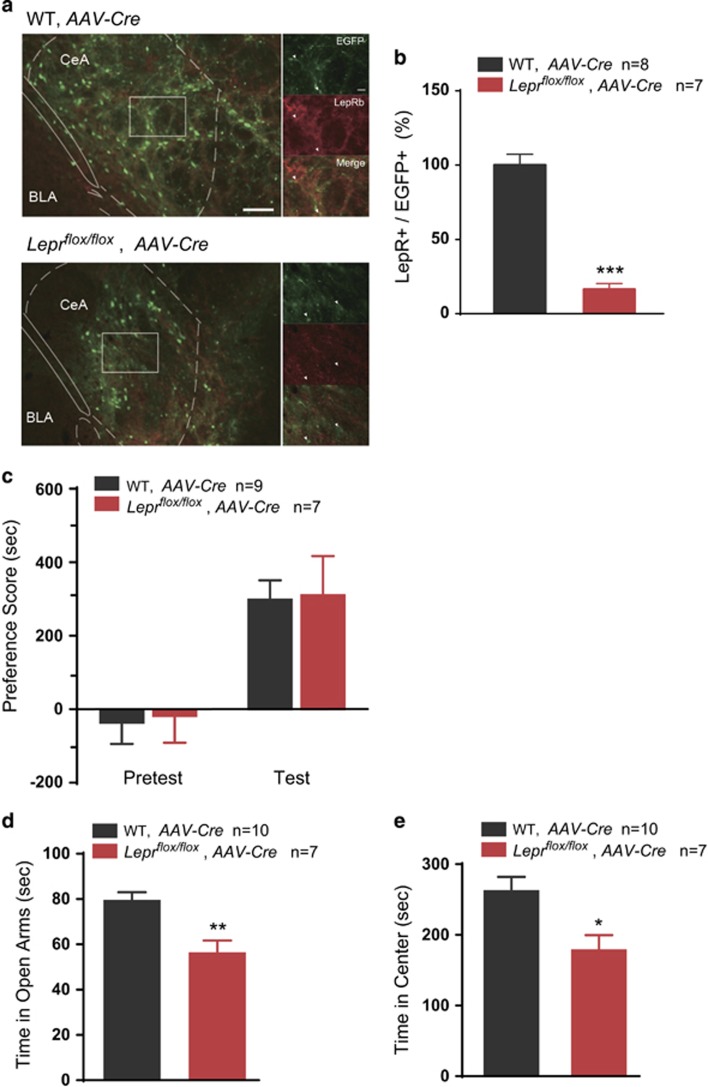
Leptin signaling in CeA is critical for anxiety but not cocaine-conditioned reward. (**a**) Representative images of the *AAV-CAG-EGFP-T2A-Cre*-infected CeA from *Lepr*^*flox/flox*^ mice and WT littermates. EGFP: green; LepR: red; arrows indicate the EGFP^+^ cells. Scale bar, 100μm (low-magnification images) and 20 μm (high-magnification images). (**b**) Quantification of the percentage of LepR-positive cells in the EGFP^+^ population in the CeA of virus-infected *Lepr*^*flox/flox*^ mice and WT mice. (**c**) Quantification of the place preference scores of *Lepr*^*flox/flox*^ and WT mice in the pre-test and test sessions. (**d**) Time spent in the open arms of the elevated plus maze test in the *Lepr*^*flox/flox*^ and WT mice. (**e**) Time in the center zone of the open-field test in the *Lepr*^*flox/flox*^ and WT mice. The data are presented as the mean±s.e.m. *P<0.05, **P<0.01, ***P<0.001. CeA, central amygdala; LepR, leptin receptor; WT, wild-type.

The CeA, which mediates stress-related processes, is also innervated by VTA transmission.^29^ *Lepr*^*flox/flox*^ mice and their WT littermates received bilateral infusion of *AAV-CAG-EGFP-T2A-Cre* into the CeA (Figure 4a), and the knockdown efficiency of the LepR in the CeA was evaluated by immunostaining (Student's *t*-test, *t*(13)=9.665, *P*<0.001; Figure 4b). In the cocaine-CPP test, there was no significant difference in the preference scores between *Lepr*^*flox/flox*^ mice and their WT littermates (two-way ANOVA, treatment × genotype: F(1, 14)=0.00187, *P*=0.966. WT vs *Lepr*^*flox/flox*^ within test, *P*=0.905; Figure 4c).

In the elevated plus maze test, the *Lepr*^*flox/flox*^ mice injected with *AAV-CAG-eGFP-T2A-Cre* in the CeA spent significantly less time in the open arms than the WT littermates (Student's *t*-test, *t*(15)=3.441, *P*=0.0036; Figure 4d). In the open-field test, *Lepr*^*flox/flox*^ mice spent significantly less time in the center zone (*t*(15)=2.774, *P*=0.0142; Figure 4e) and tended to exhibit less entries into the center zone (*t*(15)=2.108, *P*=0.0536; Supplementary Figure S4E). Although the total distance travelled was not different between the two groups (*t*(14)=0.5866, *P*=0.5668; Supplementary Figure S4F). These data suggest that leptin signaling in the CeA is not essential for cocaine-conditioned reward, while it is involved in the regulation of the negative emotional state.

### Upregulation of leptin signaling in the VTA or NAc inhibits cocaine-conditioned reward

**Figure 5:**
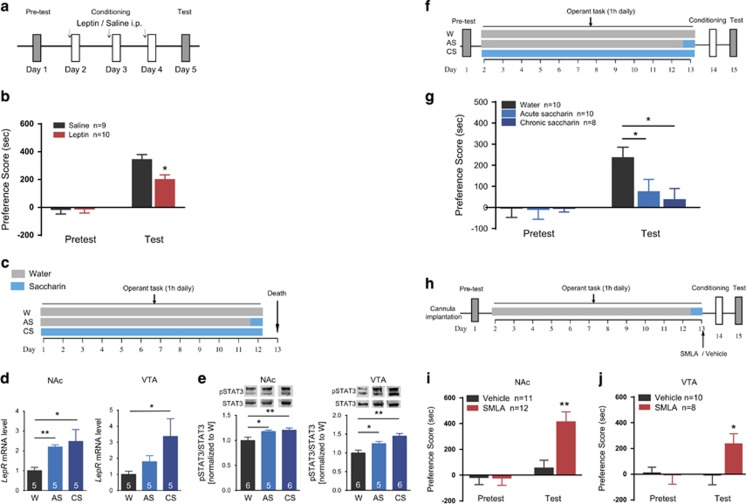
Upregulated leptin signaling reduces cocaine-CPP. (**a**, **b**) Mice received injection of saline or leptin (1 mg kg^−1^ i.p.) 30 min before conditioning, and the quantification of the place preference scores in the pre-test and test sessions is shown. (**c**) Schematic of the saccharin reward experimental schedule. Three groups of mice were confined to the apparatus for a 1-h operant task for 12 days to receive water (W) or saccharin daily (CS) or saccharin only on the last session (AS). The mice were decapitated 24 h after the last session. (**d**, **e**) The levels of *LepR* mRNA (**d**), pSTAT3 protein and total STAT3 (**e**) in the NAc and VTA were detected. Images are representative of similar results from five to six independent individuals. (**f**) Schematic of the operant task and cocaine-CPP schedule. Three groups of mice (W, AS and CS) performed a 1 h operant task for 12 days, and 24 h after the last session, each mouse received cocaine-context conditioning learning. Place preference was examined 24 h later. (**g**) Quantification of the place preference scores of the three groups of mice in the pre-test and test sessions. (**h**) Schematic of the modified cocaine-CPP schedule. SMLA (500 ng) or vehicle was infused into the NAc or VTA 30 min after saccharin administration, and the mice received cocaine-context conditioning 24 h later. (**i**, **j**) Quantification of the place preference scores of mice receiving SMLA (500 ng) infusion into the NAc (**i**) or VTA (**j**) after saccharin administration in the pre-test and test sessions. The data are presented as the mean±s.e.m. *P<0.05, **P<0.01. CPP, conditioned place preference; NAc, nucleus accumbens; SMLA, superactive mouse leptin antagonist; VTA, ventral tegmental area.

Because downregulation of leptin signaling increased cocaine-CPP, we then assessed whether upregulation of leptin signaling might have an opposite effect. Mice intraperitoneally injected with leptin during the cocaine-context conditioning sessions (Figure 5a) showed a decreased preference score (two-way ANOVA, treatment × genotype: F(1, 17)=4.684, *P*=0.038. Saline vs leptin within test, *P*=0.044; Figure 5b), suggesting that the activation of LepR decreased cocaine-CPP, which was consistent with the previous result that injection of exogenous leptin in the test session inhibited the establishment of cocaine-CPP in mice.^15^

We then measured the mesolimbic *LepR* mRNA and phosphorylated STAT3 levels in mice receiving saccharin to investigate whether palatable food reward affects mesolimbic LepR signaling. WT mice were divided into three groups, assigned to water (W), acute saccharin (AS) or chronic saccharin (CS). Each group of mice was daily confined to the apparatus for 1-h to assess water or saccharin intake (Figure 5c). The levels of *LepR* mRNA and pSTAT3 protein in the NAc and VTA were assessed 24 h after the last saccharin or water exposure. Saccharin administration significantly increased *LepR* mRNA levels in the NAc (Student's *t*-test, AS: *t*(8)=5.943, *P*=0.0003; CS: *t*(8)=2.375, *P*=0.0449 vs W) and VTA (AS: *t*(8)=1.811, *P*=0.1077; CS: *t*(8)=2.406, *P*=0.0428 vs W; Figure 5d), and both acute and chronic saccharin administration significantly induced activation of leptin signaling in the NAc and VTA as the level of pSTAT3 protein increased when compared with that of control (Student's *t*-test, NAc: AS: *t*(10)=2.280, *P*=0.0486; CS: *t*(10)=2.572, *P*=0.0278 vs W; VTA: AS: *t*(7)=2.248, *P*=0.0418; CS: *t*(7)=4.320, *P*=0.0035 vs W; Figure 5e). These data demonstrated that saccharin exposure upregulated leptin signaling in the NAc and VTA. Parallel with the upregulation of leptin signaling, cocaine-CPP was nearly abolished in mice from the AS and CS groups (two-way ANOVA, AS: treatment × group: F(1, 18)=2.499, *P*=0.129. AS vs W within test, *P*=0.026; CS: treatment × group: F(1, 17)=6.613, *P*=0.015. CS vs W within test, *P*=0.001; Figures 5f and g). To assess whether the upregulation of LepR signaling in the VTA and NAc caused by saccharin exposure affects cocaine-CPP, we injected SMLA into the NAc or VTA of mice in the AS group to inhibit the upregulated leptin signaling after saccharin exposure and performed cocaine conditioning 24 h later. The result showed that the cocaine-CPP eliminated by saccharin reward was restored by inhibition of LepR signaling (two-way ANOVA, NAc: treatment × genotype: F(1, 21)=7.954, *P*=0.007. Vehicle vs SMLA within test, *P*=0.008; VTA: treatment × genotype: F(1, 16)=4.646, *P*=0.039. Vehicle vs SMLA within test, *P*=0.044; Figures 5i and j). These results indicate that upregulation of leptin signaling decreases cocaine-CPP and support the notion that leptin signaling is a negative regulator of cocaine-conditioned reward.

## Discussion

Leptin has been shown to be involved in energy homeostasis and stress;^30^ however, emerging evidence has shown that structural and functional changes in the mesolimbic reward system are associated with reward-related behaviors, which involve hormonal regulators such as leptin, ghrelin and insulin.^31, 32^ In this study, the effects of endogenous leptin signaling on cocaine reward-associated behaviors and anxiety were detected. We found that downregulation of leptin signaling in the VTA and NAc enhanced the acquisition of cocaine-CPP, while upregulation of leptin signaling induced the opposite effect. These results indicate that leptin signaling, a natural reward regulator, negatively regulates cocaine-conditioned reward.

The VTA, one of the most important nuclei of the reward system, contains dopaminergic neurons innervating the NAc, CeA, prefrontal cortex and hippocampus as well as other regions. Previous studies in rats have shown that infusion of leptin into the VTA inhibits the activity of dopamine neurons and food intake, and knockdown of LepR in the VTA induced by AAV*-shLEPR* increases food intake and preference for sucrose.^33^ Exogenous leptin infusion into the VTA attenuates the rewarding effects of cocaine in a rat self-administration task, and infusion of leptin or the LepR agonist into the VTA blocks the establishment of cocaine-CPP,^16^ while a LepR antagonist fails to have an effect, suggesting the LepR antagonist does not increase the effectiveness of cocaine after conditioning. Our results showed that downregulating leptin signaling by i.c.v. infusion of the LepR antagonist SMLA promoted the consolidation (Supplementary Figure S1A) as well as the acquisition (Figure 1d) of cocaine-conditioned memory. Here we used an AAV-delivered Cre/loxP system to achieve the specific knockdown of the LepR in the VTA of adult mice. Our results showed that downregulation of the endogenous LepR in the VTA enhanced the effect of cocaine with no significant effects on anxiety in the elevated plus maze test, although there was a trend for a reduction in the time spent in the center zone in the open-field test, which was consistent with the recent publication by Liu *et al.*^32^ The HPLC analysis detected increased dopamine levels in the NAc after knockdown of the LepR in the VTA (Figure 2d), suggesting LepRs in the VTA are required for the function of dopaminergic axons innervating the NAc. Specific knockdown of LepRs in the dopaminergic neurons in the VTA using *DA-Cre* or *TH-Cre* mice could be used to better identify the neuronal types involved in the modulation of cocaine-conditioned reward mediated by leptin signaling.

The NAc receives dopaminergic projections from the VTA, and leptin suppresses the basal and feeding-evoked extracellular dopamine concentration in the NAc and inhibits food intake in rats.^34, 35^ In the present study, we examined the effects of endogenous LepR downregulation on cocaine-CPP. There are numerous types of neurons in the NAc, which induce many distinct and complex effects. Activation of D1-dopamine receptor (D1R)-positive medium spiny neurons during cocaine exposure enhances the development of cocaine-CPP, while activation of D2R-positive medium spiny neurons diminishes cocaine-CPP.^36^ Diet-induced obesity downregulates the level of striatal D2Rs,^37^ and knockdown of striatal D2Rs accelerates the development of addiction-like reward deficits in obese rats.^38^ Mice lacking leptin systemically show decreased D2R binding in the striatum and increased dopamine activity in reward-related brain regions including the NAc,^28^ and activation of the LepR inhibits food intake via the D2R indirect pathway.^39^ These previous results imply that LepRs are involved in the effect of D2R activation. Here we found that impaired cocaine-CPP upon D2R activation in the NAc was eliminated when LepRs in the NAc core were deleted, which suggests that LepRs are required for the D2R-induced inhibitory effects of cocaine-CPP. The function of leptin signaling in the D1R- or D2R-positive neurons in the NAc will be assessed in the future using Cre-expressing mice and might help elucidate the mechanism of leptin signaling affecting cocaine-CPP.

It has been shown that some compartments of amygdala, especially CeA, code reward-related value and affective significance to regulate reward learning and addiction.^40^ LepR-positive neurons innervate cocaine- and amphetamine-regulated transcript neurons in CeA,^41^ and intra-amygdala infusion of leptin facilitates the extinction of conditioned fear responses.^42^ Here we found that specific deletion of LepR in the CeA of adult mice increased the anxiety level while had no effect on the cocaine-CPP, suggesting that leptin signaling in the CeA might be involved in the affective evaluation, whereas is not essential to the cocaine-conditioned rewards.

Chronic exposure to high-caloric food induces obesity and reduces protein levels of D2R, CREB^43^ as well as D2R mRNA^44^ in the striatum, and also induced functional synaptic changes associated with addictive behavior,^45^ indicating food reward changes the homeostasis of reward system. Consumption of palatable food including sweet or fatty foods accelerates habitual control of behavior dependent on activation of the dorsolateral striatum,^46^ and enhances cocaine-induced locomotor sensitization which is associated with increased motivation for rewards and reward paired cues.^47^ Recent work showed that cocaine, morphine and chocolate of the same valence activate largely overlapping neural ensembles in the NAc.^48^ We found that either acute or chronic acquisition of saccharin impaired cocaine-CPP, and significantly upregulated the leptin signaling in the VTA and NAc. Inhibition of the upregulated leptin signaling by SMLA rescued the deficit of cocaine-CPP induced by saccharin reward. These results support the notion that leptin signaling is a negative regulator in the effect of addictive drugs and might also to be involved in mediating the inhibitory effect of natural reward upon drug reward.

## References

[bib1] McEwen BS, Gray JD, Nasca C. 60 YEARS OF NEUROENDOCRINOLOGY: redefining neuroendocrinology: stress, sex and cognitive and emotional regulation. J Endocrinol 2015; 226: T67–T83. 25934706 10.1530/JOE-15-0121 PMC4515381

[bib2] Sinha R, Jastreboff AM. Stress as a common risk factor for obesity and addiction. Biol Psychiatry 2013; 73: 827–835. 23541000 10.1016/j.biopsych.2013.01.032 PMC3658316

[bib3] Hildebrandt T, Greif R. Stress and addiction. Psychoneuroendocrinology 2013; 38: 1923–1927. 23849597 10.1016/j.psyneuen.2013.06.017 PMC3773022

[bib4] Haass-Koffler CL, Aoun EG, Swift RM, de la Monte SM, Kenna GA, Leggio L. Leptin levels are reduced by intravenous ghrelin administration and correlated with cue-induced alcohol craving. Transl Psychiatry 2015; 5: e646. 26418274 10.1038/tp.2015.140 PMC5545639

[bib5] Suchankova P, Yan J, Schwandt ML, Stangl BL, Caparelli EC, Momenan R et al. The glucagon-like peptide-1 receptor as a potential treatment target in alcohol use disorder: evidence from human genetic association studies and a mouse model of alcohol dependence. Transl Psychiatry 2015; 5: e583. 26080318 10.1038/tp.2015.68 PMC4490279

[bib6] Kenny PJ. Common cellular and molecular mechanisms in obesity and drug addiction. Nat Rev Neurosci 2011; 12: 638–651. 22011680 10.1038/nrn3105

[bib7] Zhang Y, Proenca R, Maffei M, Barone M, Leopold L, Friedman JM. Positional cloning of the mouse obese gene and its human homologue. Nature 1994; 372: 425–432. 7984236 10.1038/372425a0

[bib8] Friedman JM, Halaas JL. Leptin and the regulation of body weight in mammals. Nature 1998; 395: 763–770. 9796811 10.1038/27376

[bib9] Dhillon H, Zigman JM, Ye C, Lee CE, McGovern RA, Tang V et al. Leptin directly activates SF1 neurons in the VMH, and this action by leptin is required for normal body-weight homeostasis. Neuron 2006; 49: 191–203. 16423694 10.1016/j.neuron.2005.12.021

[bib10] Wang X, Zhang D, Lu XY. Dentate gyrus-CA3 glutamate release/NMDA transmission mediates behavioral despair and antidepressant-like responses to leptin. Mol Psychiatry 2015; 20: 509–519. 25092243 10.1038/mp.2014.75 PMC4362753

[bib11] Fernandes MF, Matthys D, Hryhorczuk C, Sharma S, Mogra S, Alquier T et al. Leptin suppresses the rewarding effects of running via STAT3 signaling in dopamine neurons. Cell Metab 2015; 22: 741–749. 26341832 10.1016/j.cmet.2015.08.003

[bib12] Balland E, Cowley MA. New insights in leptin resistance mechanisms in mice. Front Neuroendocrinol 2015; 39: 59–65. 26410445 10.1016/j.yfrne.2015.09.004

[bib13] Fulton S, Pissios P, Manchon RP, Stiles L, Frank L, Pothos EN et al. Leptin regulation of the mesoaccumbens dopamine pathway. Neuron 2006; 51: 811–822. 16982425 10.1016/j.neuron.2006.09.006

[bib14] Lim G, Kim H, McCabe MF, Chou CW, Wang S, Chen LL et al. A leptin-mediated central mechanism in analgesia-enhanced opioid reward in rats. J Neurosci 2014; 34: 9779–9788. 25031415 10.1523/JNEUROSCI.0386-14.2014 PMC4099551

[bib15] Morales L, Del Olmo N, Valladolid-Acebes I, Fole A, Cano V, Merino B et al. Shift of circadian feeding pattern by high-fat diets is coincident with reward deficits in obese mice. PloS One 2012; 7: e36139. 22570696 10.1371/journal.pone.0036139 PMC3343034

[bib16] You ZB, Wang B, Liu QR, Wu Y, Otvos L, Wise RA. Reciprocal inhibitory interactions between the reward-related effects of leptin and cocaine. Neuropsychopharmacology 2015; 41: 1024–1033. 26243270 10.1038/npp.2015.230 PMC4748427

[bib17] Tartaglia LA, Dembski M, Weng X, Deng N, Culpepper J, Devos R et al. Identification and expression cloning of a leptin receptor, OB-R. Cell 1995; 83: 1263–1271. 8548812 10.1016/0092-8674(95)90151-5

[bib18] Guo M, Lu Y, Garza JC, Li Y, Chua SC, Zhang W et al. Forebrain glutamatergic neurons mediate leptin action on depression-like behaviors and synaptic depression. Transl Psychiatry 2012; 2: e83. 22408745 10.1038/tp.2012.9 PMC3298113

[bib19] Guo M, Huang TY, Garza JC, Chua SC, Lu XY. Selective deletion of leptin receptors in adult hippocampus induces depression-related behaviours. Int J Neuropsychopharmacol 2013; 16: 857–867. 22932068 10.1017/S1461145712000703 PMC3612133

[bib20] Liu J, Perez SM, Zhang W, Lodge DJ, Lu XY. Selective deletion of the leptin receptor in dopamine neurons produces anxiogenic-like behavior and increases dopaminergic activity in amygdala. Mol Psychiatry 2011; 16: 1024–1038. 21483433 10.1038/mp.2011.36 PMC3432580

[bib21] Shpilman M, Niv-Spector L, Katz M, Varol C, Solomon G, Ayalon-Soffer M et al. Development and characterization of high affinity leptins and leptin antagonists. J Biol Chem 2011; 286: 4429–4442. 21119198 10.1074/jbc.M110.196402 PMC3039357

[bib22] Kay K, Parise EM, Lilly N, Williams DL. Hindbrain orexin 1 receptors influence palatable food intake, operant responding for food, and food-conditioned place preference in rats. Psychopharmacology 2014; 231: 419–427. 23978908 10.1007/s00213-013-3248-9 PMC3946976

[bib23] Figlewicz DP, Higgins MS, Ng-Evans SB, Havel PJ. Leptin reverses sucrose-conditioned place preference in food-restricted rats. Physiol Behav 2001; 73: 229–234. 11399316 10.1016/s0031-9384(01)00486-3

[bib24] Scott MM, Lachey JL, Sternson SM, Lee CE, Elias CF, Friedman JM et al. Leptin targets in the mouse brain. J Comp Neurol 2009; 514: 518–532. 19350671 10.1002/cne.22025 PMC2710238

[bib25] Kenny PJ. Reward mechanisms in obesity: new insights and future directions. Neuron 2011; 69: 664–679. 21338878 10.1016/j.neuron.2011.02.016 PMC3057652

[bib26] Davis JF, Choi DL, Schurdak JD, Fitzgerald MF, Clegg DJ, Lipton JW et al. Leptin regulates energy balance and motivation through action at distinct neural circuits. Biol Psychiatry 2011; 69: 668–674. 21035790 10.1016/j.biopsych.2010.08.028 PMC3058141

[bib27] Fulton S, Woodside B, Shizgal P. Modulation of brain reward circuitry by leptin. Science 2000; 287: 125–128. 10615045 10.1126/science.287.5450.125

[bib28] Pfaffly J, Michaelides M, Wang GJ, Pessin JE, Volkow ND, Thanos PK. Leptin increases striatal dopamine D2 receptor binding in leptin-deficient obese (ob/ob) mice. Synapse 2010; 64: 503–510. 20175225 10.1002/syn.20755 PMC2873172

[bib29] Kalivas PW, McFarland K. Brain circuitry and the reinstatement of cocaine-seeking behavior. Psychopharmacology 2003; 168: 44–56. 12652346 10.1007/s00213-003-1393-2

[bib30] Wauman J, Tavernier J. Leptin receptor signaling: pathways to leptin resistance. Front Biosci (Landmark Ed) 2011; 16: 2771–2793. 21622208 10.2741/3885

[bib31] Vengeliene V. The role of ghrelin in drug and natural reward. Addict Biol 2013; 18: 897–900. 24283979 10.1111/adb.12114

[bib32] Liu J, Guo M, Lu XY. Leptin/LepRb in the ventral tegmental area mediates anxiety-related behaviors. Int J Neuropsychopharmacol 2015; 19: pyv115. 26438799 10.1093/ijnp/pyv115 PMC4772826

[bib33] Figlewicz DP, Evans SB, Murphy J, Hoen M, Baskin DG. Expression of receptors for insulin and leptin in the ventral tegmental area/substantia nigra (VTA/SN) of the rat. Brain Res 2003; 964: 107–115. 12573518 10.1016/s0006-8993(02)04087-8

[bib34] Hommel JD, Trinko R, Sears RM, Georgescu D, Liu ZW, Gao XB et al. Leptin receptor signaling in midbrain dopamine neurons regulates feeding. Neuron 2006; 51: 801–810. 16982424 10.1016/j.neuron.2006.08.023

[bib35] Krugel U, Schraft T, Kittner H, Kiess W, Illes P. Basal and feeding-evoked dopamine release in the rat nucleus accumbens is depressed by leptin. Eur J Pharmacol 2003; 482: 185–187. 14660021 10.1016/j.ejphar.2003.09.047

[bib36] Lobo MK, Covington HE 3rd, Chaudhury D, Friedman AK, Sun H, Damez-Werno D et al. Cell type-specific loss of BDNF signaling mimics optogenetic control of cocaine reward. Science 2010; 330: 385–390. 20947769 10.1126/science.1188472 PMC3011229

[bib37] Kenny PJ, Voren G, Johnson PM. Dopamine D2 receptors and striatopallidal transmission in addiction and obesity. Curr Opin Neurobiol 2013; 23: 535–538. 23726225 10.1016/j.conb.2013.04.012 PMC3799805

[bib38] Johnson PM, Kenny PJ. Dopamine D2 receptors in addiction-like reward dysfunction and compulsive eating in obese rats. Nat Neurosci 2010; 13: 635–641. 20348917 10.1038/nn.2519 PMC2947358

[bib39] Billes SK, Simonds SE, Cowley MA. Leptin reduces food intake via a dopamine D2 receptor-dependent mechanism. Mol Metab 2012; 1: 86–93. 24024122 10.1016/j.molmet.2012.07.003 PMC3757652

[bib40] Sangha S, Chadick JZ, Janak PH. Safety encoding in the basal amygdala. J Neurosci 2013; 33: 3744–3751. 23447586 10.1523/JNEUROSCI.3302-12.2013 PMC6619315

[bib41] Leshan RL, Opland DM, Louis GW, Leinninger GM, Patterson CM, Rhodes CJ et al. Ventral tegmental area leptin receptor neurons specifically project to and regulate cocaine- and amphetamine-regulated transcript neurons of the extended central amygdala. J Neurosci 2010; 30: 5713–5723. 20410123 10.1523/JNEUROSCI.1001-10.2010 PMC2864009

[bib42] Wang W, Liu SL, Li K, Chen Y, Jiang B, Li YK et al. Leptin: a potential anxiolytic by facilitation of fear extinction. CNS Neurosci Ther 2015; 21: 425–434. 25645604 10.1111/cns.12375 PMC6495693

[bib43] Adams WK, Sussman JL, Kaur S, D'Souza AM, Kieffer TJ, Winstanley CA. Long-term, calorie-restricted intake of a high-fat diet in rats reduces impulse control and ventral striatal D receptor signaling: two markers of addiction vulnerability. Eur J Neurosci 2015; 42: 3095–3104. 26527415 10.1111/ejn.13117

[bib44] Robinson MJ, Burghardt PR, Patterson CM, Nobile CW, Akil H, Watson SJ et al. Individual differences in cue-induced motivation and striatal systems in rats susceptible to diet-induced obesity. Neuropsychopharmacology 2015; 40: 2113–2123. 25761571 10.1038/npp.2015.71 PMC4613617

[bib45] Brown RM, Kupchik YM, Spencer S, Garcia-Keller C, Spanswick DC, Lawrence AJ et al. Addiction-like synaptic impairments in diet-induced obesity. Biol Psychiatry 2015; S0006-3223(0015)00996-00998; doi:10.1016/j.biopsych.2015.11.019. 10.1016/j.biopsych.2015.11.019 PMC4889544 26826876

[bib46] Furlong TM, Jayaweera HK, Balleine BW, Corbit LH. Binge-like consumption of a palatable food accelerates habitual control of behavior and is dependent on activation of the dorsolateral striatum. J Neurosci 2014; 34: 5012–5022. 24695718 10.1523/JNEUROSCI.3707-13.2014 PMC6802720

[bib47] Oginsky MF, Maust JD, Corthell JT, Ferrario CR. Enhanced cocaine-induced locomotor sensitization and intrinsic excitability of NAc medium spiny neurons in adult but not in adolescent rats susceptible to diet-induced obesity. Psychopharmacology 2016; 233: 773–784. 26612617 10.1007/s00213-015-4157-x PMC4752900

[bib48] Xiu J, Zhang Q, Zhou T, Zhou TT, Chen Y, Hu H. Visualizing an emotional valence map in the limbic forebrain by TAI-FISH. Nat Neurosci 2014; 17: 1552–1559. 25242305 10.1038/nn.3813

