# Supplementary material for: Mesolimbic leptin signaling negatively regulates cocaine-conditioned reward
Source: Transl Psychiatry. 2016 Dec 6;6(12):e972–. doi: 10.1038/tp.2016.223 (PMC5315559; doi:10.1038/tp.2016.223)
Supplement: Supplementary Figures [file tp2016223x1.docx]

# SUPPLEMENTARY MATERIAL

#
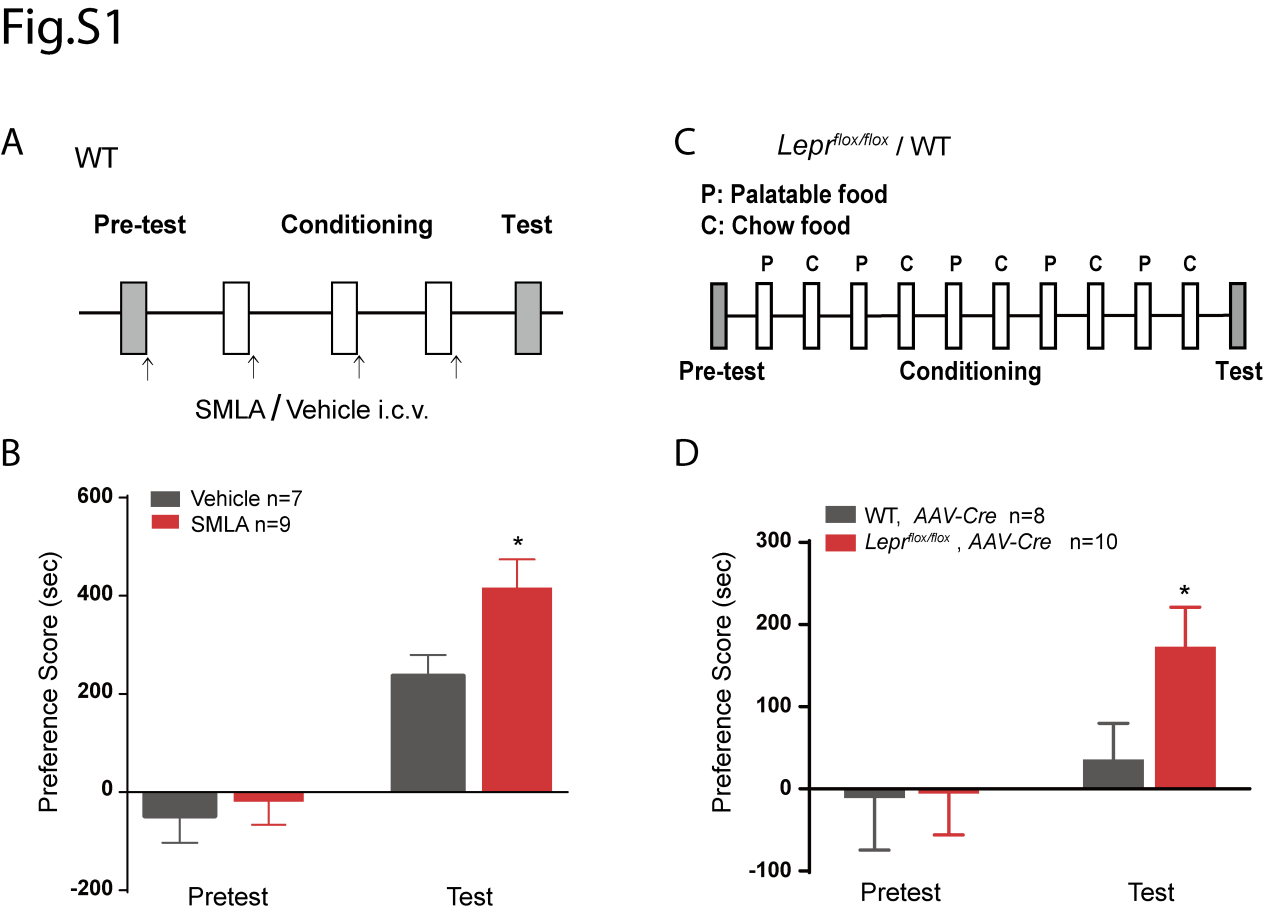


# Fig. S1. The effect of specific downregulated eptin signaling on the cocaine-CPP and palatable food-induced CPP

(A) Schematic of the experimental schedule. WT mice received an infusion of ACSF or SMLA (500 ng, i.c.v.) immediately after pre-test and each conditioning sessions. (B) Quantification of the place preference scores in the pre-test and test sessions. (C-D) *Leprflox/flox* and WT mice were injected with AAV*-CAG-EGFP-T2A-Cre* into the VTA. (C) Schematic of the palatable food-induced CPP experimental schedule. (D) Quantification of the place preference scores of *Leprflox/flox* and WT mice in the pre-test and test sessions. The data are presented as the mean ± s.e.m *P < 0.05.


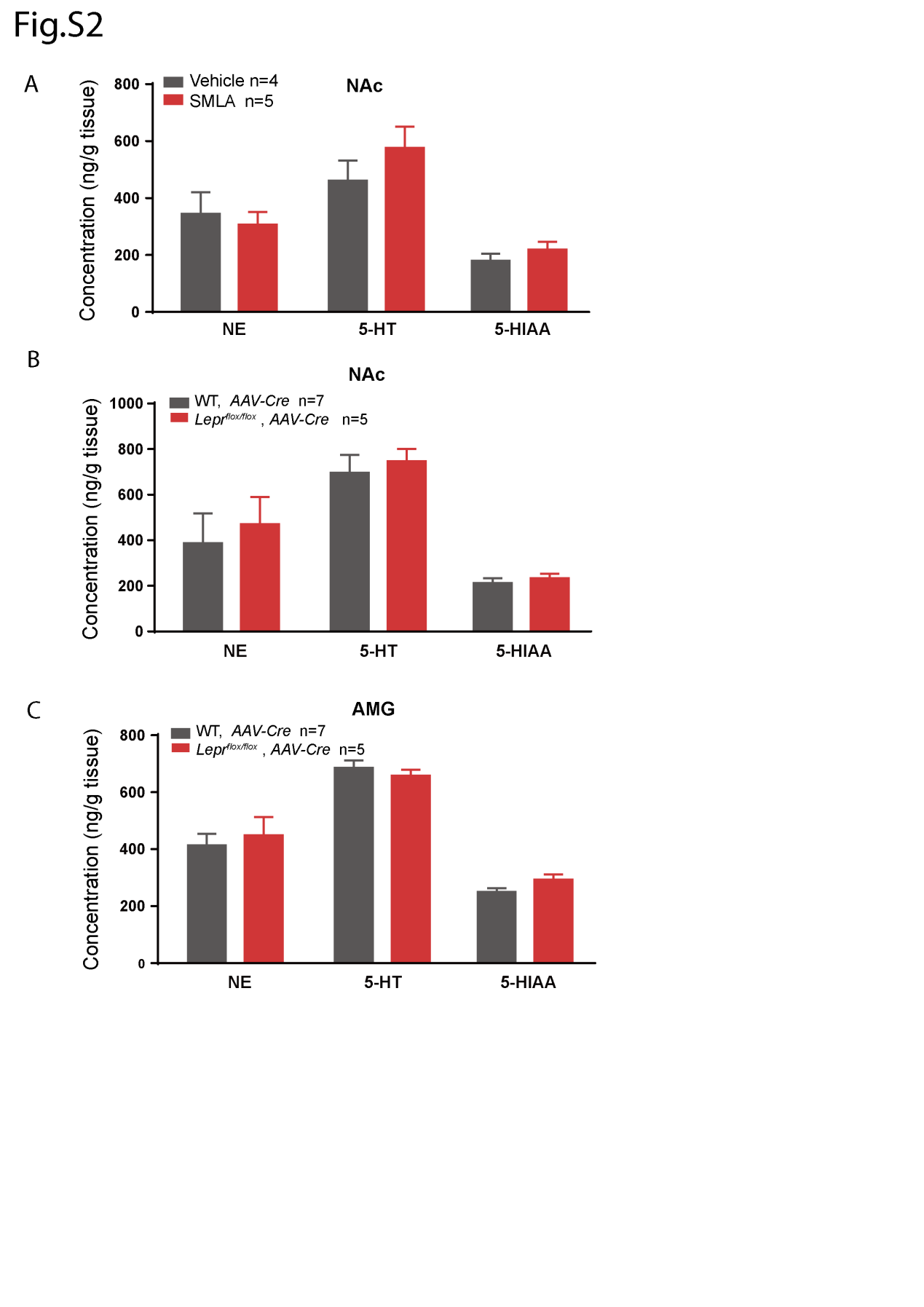


# Fig. S2. Level of cocaine-induced monoamine neurotransmitters after downregulation of leptin signaling

(A) Concentration of cocaine-induced NE, 5-HT and 5-HIAA in the NAc of mice receiving an infusion of SMLA or ACSF i.c.v.. (B-C) Concentration of the cocaine-induced NE, 5-HT and 5-HIAA in the NAc (B) and AMG (C) of *Leprflox/flox* and WT mice infected with AAV-Cre in VTA. The data are presented as the mean ± s.e.m.


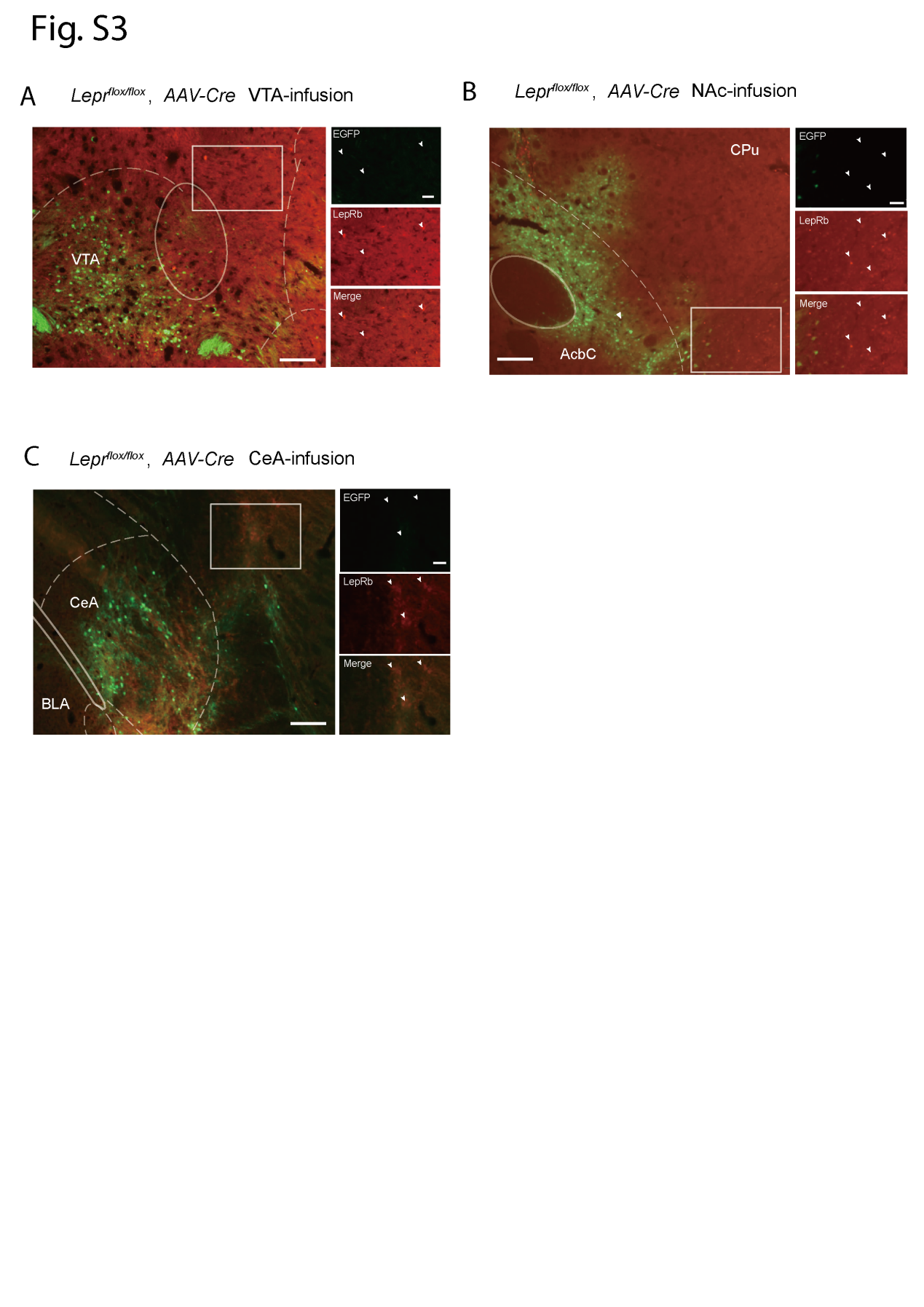


# Fig. S3. The expression of LepRs in brain regions nearby the AAV-Cre injected VTA, NAc Core and CeA

Representative images of the *AAV-CAG-EGFP-T2A-Cre* infected VTA, NAc and CeA from the *Leprflox/flox* mice. High-magnification images showed the expression of LepRs nearby the regions infected by virus. EGFP: green; LepR: red; Arrows indicate the LepR+ cells. Scale bar, 100μm (low-magnification images) and 50 μm (high-magnification images).


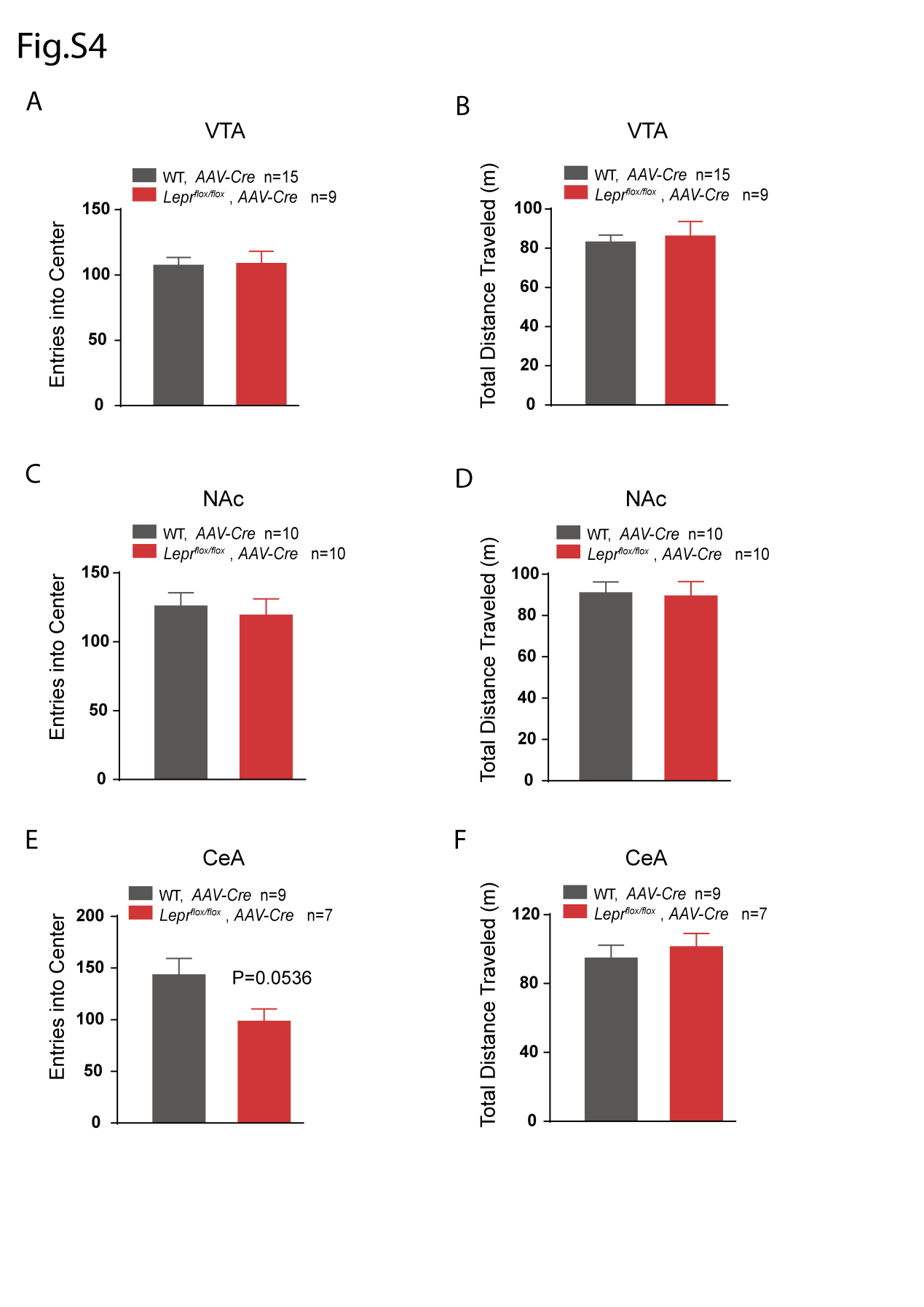


# Fig. S4. The effect of specific downregulated LepR in VTA, NAc or CeA on the anxiety level and locomotor activity of mice

# The open field test in *Leprflox/flox* and WT mice infected with AAV*-CAG-EGFP-T2A-Cre* into the VTA (A, B), NAc (C, D) or CeA (E, F). Entries into center (A, C, E), and the total distance travelled (B, D, F) were measured for 30 min. The data are presented as the mean ± s.e.m. *P< 0.05.

**SUPPLEMENTARY METHODS**

# Open field tests

An activity monitor system (43.2 cm length × 43.2 cm width × 30.5 cm height, Med-Associates, St. Albans, VT, USA) was used to detect horizontal movement. In brief, this system uses paired sets of photo beams to detect movement of mice in the open field, and movements recorded as beam breaks. Each mouse was placed in the center of the open field and allowed to explore freely for predetermined time. The total distance traveled, the entries and the time spent in the center zone were recorded.

# Elevated plus maze test

The elevated plus maze consisted of a center platform and four arms (34.5 cm length × 6.3 cm width × 19.5 cm height) placed 75 cm above the floor. Two of the arms had 20 cm high dark walls (closed arms), and two had 0.8 cm high ledges (open arms). The arms were angled at 90° to each other. The apparatus was placed in a quiet and dimmed room. Mice were placed in the center, and their behaviors were recorded for 5 min with a camera located above the maze. EthoVision XT 8.5 video tracking program (Leesburg, VA, USA) was used to track the location, velocity and movement of head, body and tail. Time spent and entries in the different compartments (closed and open arms) were assessed. The arms were cleaned between each test to ensure the absence of olfactory cues.

# RNA extraction and real-time PCR analysis

Mice were decapitated, and the brains were removed immediately. The NAc, VTA, and CeA were dissected within 5 min in iced PBS. The intended stereotaxic coordinates were as follows: NAc: from Bregma 1.54 mm to 0.86 mm; CeA: from Bregma -1.06 mm to -1.82 mm; VTA: from Bregma -2.92 mm to -3.40 mm, and frozen in liquid nitrogen and stored at –70 °C until the extraction. Total RNA was extracted from tissues using the TRIzol® Reagent (Thermo Fisher Scientific Inc, Waltham, MA, USA) according to the manufacturer’s instructions. The RT-PCR were performed with the Superscript First-Strand Synthesis system and the Power SYBR Green PCR Master Mix (TAKARA, Shiga, Japan) using the Eppendorf Mastercycler ep gradient S PCR System (Eppendorf, Germany). The primers for RT-PCR were as follows: 5'-TGG TCC CAG CAG CTA TGG T-3' and 5'-ACC CAG AGA AGT TAG CAC TGT-3' for *LepR*, 5'-GTG GAG TCA TAC TGG AAC ATG TAG-3' and 5'-AAT GGT GAAGGT CGG TGT G-3' for *GAPDH*. *LepR* mRNA expression was normalized to the internal control *GAPDH*.

# Western blotting

Brain tissues were lysed in RIPA buffer (50 mM Tris, pH 7.4，150 mM NaCl，1% NP-40， 0.5% sodium deoxycholate，0.1% SDS，and protein inhibitors). Antibodies of rabbit anti-phospho-STAT3 (Tyr705) and mouse anti-STAT3 were from Cell Signaling Technology (Danvers, MA, USA), IRDye 800CW-conjugated or 700CW-conjugated antibody were from Rockland Biosciences (Gilbertsville, PA, USA). The infrared fluorescence images were obtained with the Odyssey infrared imaging system (Li-Cor Bioscience, Lincoln, NE, USA).

# Leptin injection

Recombinant murine leptin (PeproTech, Rocky Hill, NJ, USA) was dissolved into 0.1 mg/μl in saline, and injected (1 mg/kg, i.p.) 30 min before cocaine conditioning. Control mice were administered with equal volume of saline.

# Immunohistochemistry

Mice were anesthetized with choral hydrate and perfused with saline followed by 4% paraformaldehyde in 0.1 M phosphate-buffered saline (PBS). The brains were removed, fixed in 4% paraformaldehyde overnight and subjected to dehydration in increasing sucrose solutions (20%–30%) at 4 ºC for 72 hours before slicing NAc, VTA, and amygdala. The slices were incubated in PBS solution with 3% goat serum and 0.2% Triton-X for 1 h. Then they were incubated with mouse anti-LepR (1:100 Santa Cruz Biotechnology, Dallas, Texas, USA) antibody overnight at 4 ºC. Slices were rinsed in PBS then incubated in goat anti-mouse Cy3 (Jackson Immunoresearch, West Grove, PA, USA) for 1 hour and mounted. Images were acquired on a microscope using 10× or 40 × air objectives (DP-80; Olympus, Japan).
